# Epidemiology and biological characteristics of influenza A (H4N6) viruses from wild birds

**DOI:** 10.1080/22221751.2024.2418909

**Published:** 2024-10-17

**Authors:** Xingdong Song, Jingman Tian, Minghui Li, Xiaoli Bai, Zhiguo Zhao, Jianzhong Shi, Xianying Zeng, Guobin Tian, Yuntao Guan, Hongliang Chai, Yanbing Li, Hualan Chen

**Affiliations:** a State Key Laboratory for Animal Disease Control and Prevention, Harbin Veterinary Research Institute, Chinese Academy of Agricultural Sciences, Harbin, People’s Republic of China; b College of Wildlife and Protected Area, Northeast Forestry University, Harbin, People’s Republic of China

**Keywords:** Influenza A (H4N6) virus, epidemiology, phylogeny, receptor binding property, replicative ability, pathogenicity

## Abstract

During the active surveillance, we isolated nine H4N6 subtype influenza A viruses from wild birds in China. To reveal the epidemiology and biology characteristics of H4 subtype influenza A virus from wild birds, we investigated H4 subtype viruses available in the public source, and found that the H4 viruses have been detected in at least 37 countries to date, and more than 73.6% of the viruses were from wild Anseriformes. Bayesian phylogeographic analysis showed that Mongolia worked as the important transmission centre for Eurasian lineage H4 viruses spreading. Phylogenetic analysis of HA genes indicated that global H4 influenza A viruses were divided into Eurasian and North American lineage, our nine H4N6 isolates fell into the Eurasian lineage. Recombination analysis suggested that nine H4N6 isolates underwent complex gene recombination with various subtypes of influenza A viruses and formed two genotypes. Notably, nine H4N6 isolates acquired mammalian virulence-increasing residues. Two representative H4N6 viruses possessed dual receptor binding specificity, they could efficiently replicate in MDCK and 293 T cells in vitro infection, also could cross the species barrier to infect mice directly without prior adaption in vivo experiments. These findings emphasize the public health issues represented by H4 viruses, and highlight the need to strengthen the active surveillance of H4 viruses from wild birds.

## Introduction

Avian influenza virus (AIV), a member of the Orthomyxoviridae family, is an enveloped negative-strand RNA virus with a segmented genome [1]. Based on the antigenic characteristics of two surface glycoproteins haemagglutinin (HA) and neuraminidase (NA), AIVs are classified as different subtypes [2], and 16 HA (H1 – H16) and nine NA (N1 – N9) subtypes have been described in wild aquatic birds so far [1,3]. According to the Intravenous Pathogenicity Index (IVPI > 1.2) of the virus tested in chickens or the molecular signature of cleavage site motifs with multiple basic amino acids in their HA proteins, AIVs can also be divided into highly pathogenic avian influenza virus (HPAIV) and low pathogenic avian influenza virus (LPAIV) [4,5]. HPAIV is a persistent danger to the global poultry industry and public health, while the potential threat posed by LPAIV also cannot be ignored. It is reported that certain LPAIVs are even able to cross the interspecies barrier and directly infect humans. In 1998, the first human infection with H9N2 AIV occurred in Hong Kong, China [6]. In 2010, the human case with H10N7 AIV infection was reported in Australia [7]. In 2013, China reported the first case of human infection with H10N8 and H7N9 LPAIV [8,9], respectively, and the first human case of H6N1 AIV infection was confirmed in Taiwan region in the same year [10]. Subsequently, H7N4 virus was identified in a 68-year-old woman in China in early 2018 [11]. Recently, H10N3 virus was found to cause human infection in China in 2021 [12], and a four-year-old boy was confirmed to be infected with a novel reassortant H3N8 AIV in China in 2022 [13].

The H4 subtype AIV typically induces mild clinical manifestations and low mortality rates in poultry, and is classified as an LPAIV, and it has spread widely to Asia, Europe and North America since its first isolation from a duck in Czechoslovakia in 1956 [14]. Although poultry and aquatic birds are the primary reservoirs of H4 AIVs [15], these viruses can cross species barriers to infect mammals, such as mice [16], seals [17] and swines [18,19]. Previous studies have shown that H4 AIVs can be transmitted between guinea pigs by direct contact [14], and acquire the ability to bind to human-type receptors (α−2,6-sialic acid receptors) [20]. It is worth noting that human case with H4 virus infection has not been reported so far, however, H4 AIV-specific antibodies were detected in chicken farm workers in Lebanon and United States [21,22]. Therefore, we propose that we should pay more attention to the public health threat from H4 subtype AIVs.

Our previous study showed that we isolated 36 representative H4 subtype AIVs from swab samples collected from live poultry markets in China between 2009 and 2012, and H4N6 subtype occupied the largest proportion (19 out of 36) [14]. In this study, we isolated 17 AIVs from 1017 fresh fecal samples that collected from wild bird habitats in Anhui Province, China in October, 2020, with an AIV positivity rate reached 1.6%, and H4N6 subtype accounted for more than 52.9% (9 out of 17) among the 17 viruses (Table S1). Subsequently, we investigated the epidemiology of global H4 subtype viruses, and conducted a phylodynamic analysis for H4 viruses belonging to Eurasian lineage. In addition, we further analyzed the genetic evolution, molecular characteristics, receptor binding properties, replication ability in vitro and pathogenicity in mice of H4N6 AIVs isolated from wild birds in Anhui Province, China, 2020. Our data demonstrated that the H4 viruses posed a potential threat to public health, and emphasized the need for continued monitoring of H4 influenza virus from wild birds.

## Materials and methods

### Ethics statements

Animal experiments were completed under the Guide for the Care and Use of Laboratory Animals of the Ministry of Science and Technology of the People's Republic of China, and were endorsed by the Committee on the Ethics of Animal Experiments of the Harbin Veterinary Research Institute (HVRI), Chinese Academy of Agricultural Sciences (CAAS).

### 2.2 Biosecurity

The virus isolation and experiments with live H4N6 viruses were conducted within the enhanced biosafety level 2 (BSL2+) laboratory in the HVRI, CAAS.

### 2.3 Sample collection and virus isolation

In October 2020, we collected 1017 fresh fecal samples in Caizi Lake National Wetland Park, Anhui Province, China, then they were inoculated into 10-day-old SPF chicken embryos. After incubation at 37  °C for 72 h, the allantoic fluid was collected from each egg for the HA test. Hemagglutination inhibition (HI) tests were performed using laboratory-prepared H1–H16 reference sera to determine the HA subtypes of the allantoic fluid with HA activity. NA subtypes were identified by N1–N9 specific primers [23].

### Genome sequencing

Viral RNA from HA-positive allantoic fluid was extracted using QIAamp Viral RNA Mini Kit (Qiagen, Hilden, Germany) and reverse transcribed into cDNA using specific primers. Eight gene segments of influenza virus were amplified by RT-PCR and then sequenced using Applied Biosystems DNA analyzer. Sequences were assembled with SeqMan software in DNASTAR Lasergene 7.1 package. The sequences of the primers used in the study are available from the authors upon request.

### Sequence analysis

The HA sequences of global H4 subtype viruses were retrieved from the GISAID [24] EpiFlu (http://platform.gisaid.org) database (accessed on October 8th, 2023), and the coding regions of these sequences were aligned using MAFFT v7.490 [25]. To obtain the valid sequences for time and geographical distribution statistics, duplicate and low-quality sequences were removed. The valid HA sequences and our isolates were combined into a dataset, further used to construct the phylogenetic tree using IQ-TREE v.1.6.12 [26] by maximum likelihood (ML) method. The resulting ML phylogenetic tree was visualized and annotated using Chiplot (https://www.chiplot.online). Similarly, phylogenetic analysis of our isolates was performed using the ML method provided by MEGA v7.0 software with 1000 ultrafast bootstraps. Each gene segment in the ML tree was grouped using sequence homology greater than 95% as a criterion.

### Bayesian phylodynamic analysis

In BEAST v1.10.4, a SRD06 nucleotide substitution model and an uncorrelated lognormal relaxed clock model [27] were selected to construct a maximum clade credibility (MCC) tree of HA sequences from H4 viruses in Eurasian lineage. The Markov chain Monte Carlo (MCMC) chain was run for 300 million generations, with sampling every 10,000 steps, and the convergence of the MCMC chain was assessed in Tracer v1.7.2 [28] to ensure the effective sample size (ESS)  of ≥ 200. Finally, the MCC tree was obtained using TreeAnnotator v1.10.4 with 10% burn-in and then annotated in FigTree v1.4.4. To estimate the transmission patterns of the H4 viruses in the Eurasian lineage, phylodynamic analysis was performed using an asymmetric model, with Bayesian stochastic search variable selection (BSSVS) implemented in BEAST v1.10.4 [29]. Sequences in the dataset (n = 229) were divided into 11 geographic regions and eight host categories. Bayes factor (BF) tests were carried out to provide statistical support for potential transmission routes between different geographic locations and between different hosts using spreaD3 v0.9.6 [30]. The migration pathway was considered as supported based on the combination of the BF of  ≥  3 and the posterior probability (PP) of  ≥  0.5.

### Receptor binding analysis

Two representative H4N6 isolates showed the higher viral titer in cell than the other isolates in genotype one and two, so A/little egret/Anhui/A1-156/2020(H4N6) (LG/AH/A1-156/2020(H4N6)) and A/mallard/Anhui/A9-999/2020(H4N6) (ML/AH/A9-999/2020(H4N6)) were selected for receptor binding assay. As described in previous studies, solid-phase binding tests were conducted using α−2,3-siaylglycopolymer and α−2,6-sialylglycopolymer to examine the receptor preference of viruses [31]. For two representative H4N6 isolates, chicken antisera (ML/JS/1-1-965/2023(H4N6)) was used as the primary antibody, and a horseradish peroxidase (HRP)-conjugated goat-anti-chicken antibody (Sigma-Aldrich, St. Louis, MO, USA) was used as the secondary antibody.

### Virus growth curves in vitro

Two representative H4N6 viruses were inoculated into MDCK (a canine kidney epithelial cell line) and 293 T (a human renal embryonic epithelial cell line) monolayers at a multiplicity of infection (MOI) of 0.01. One hour after infection, the cells were washed and covered with Opti-MEM, then incubated at 37 °C. The supernatants were harvested at various time points and titered in MDCK cells.

### Mouse test

Female 6-week-old BALB/c mice (Vital River Laboratories, Beijing, China) were divided into two inoculation groups (eight mice each) and one control group (five mice). Two inoculation groups were anaesthetised via isoflurane inhalation and then inoculated intranasally with 10^6.0^ 50% egg infectious dose (EID_50_) of two representative H4N6 viruses in a 50 μL volume, respectively, with control group inoculated with 50 μl PBS. On day 3 post-infection (p.i.), three mice per inoculation group were euthanized to detect viral titers in their lungs, nasal turbinates, kidneys, spleens and brains using Reed-Muench method [32]. Five mice in two inoculation groups and control group were monitored daily for body weight change until day 14 p.i..

## Results

### The epidemiology of H4 subtype viruses

**Figure 1.**
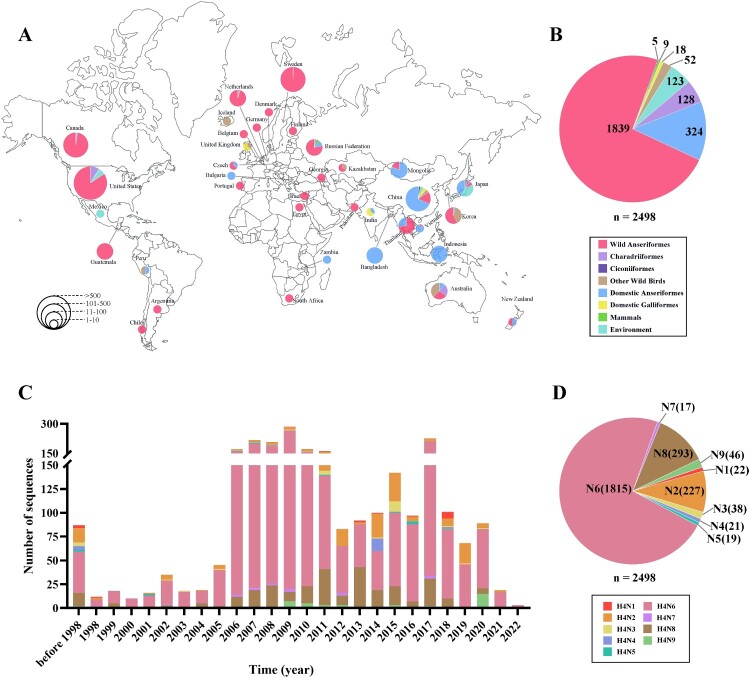
Spatial and temporal distribution of global H4 subtype viruses. (A) Global distribution of H4 subtype viruses. (B) The proportion of global H4 virus hosts. (C) The number of global H4 isolates per year. (D) The proportion of global H4 virus subtypes.

To better understand the prevalence of the H4 viruses globally, we obtained a total of 2498 HA sequences (nine isolated from wild birds in this study and 2489 from the GISAID EpiFlu database) of global H4 subtype viruses for time and geographical distribution statistics. As shown in Figure 1A, the H4 viruses were distributed in at least 37 countries, with the largest number of isolates in the United States (n = 1244), followed by Canada (n = 338), Sweden (n = 236) and China (n = 233), and more than 63.9% of isolates came from North American countries. In terms of hosts, about 73.6% of H4 viruses were isolated from wild Anseriformes (n = 1839), followed by domestic Anseriformes (n = 324; 12.9%) and Charadriiformes (n = 128; 5.1%), and more than 81.0% of H4 viruses were derived from wild birds (n = 2024) (Figure 1B). In addition, we found the number of H4 isolates was at a relatively high level from 2006 to 2020, and reached a peak of 285 in 2009 (Figure 1C). With regard to subtypes, the H4N6 subtype was the dominant subtype combination of the global H4 influenza viruses (n = 1815; 72.6%), followed by H4N8 (n = 293; 11.7%) and H4N2 (n = 227; 9.0%), while the proportions of other H4 subtype viruses, including H4N1, H4N3, H4N4, H4N5, H4N7 and H4N9, were low (Figure 1D).

We further conducted time and geographical distribution statistics on the 233 HA sequences of H4 subtype viruses in China. As shown in Figure S1 A, the H4 viruses were detected in at least 21 provinces, with the largest number of isolates in Taiwan region (n = 48), followed by Sichuan Province (n = 33), Shanghai (n = 28) and Zhejiang Province (n = 25), and more than 60.9% of isolates came from eastern China (including Shandong Province, Jiangsu Province, Anhui Province, Shanghai, Zhejiang Province, Jiangxi Province, Fujian Province and Taiwan region) (n = 142). With regard to hosts, approximately 67.3% of H4 viruses were isolated from duck (n = 157), followed by wild Anseriformes (n = 46; 19.7%), chicken (n = 10; 4.2%) and environment (n = 10; 4.2%) (Figure S1 B). In addition, we found the number of H4 isolates was at a relatively high level in three periods: 2006, 2009 – 2015 and 2019 – 2020, and reached a peak of 42 in 2015 (Figure S1 C). In terms of subtypes, the H4N6 subtype was the dominant subtype combination of H4 viruses in China (n = 108; 46.3%), followed by H4N2 (n = 83; 35.6%) and H4N8 (n = 20; 8.5%) (Figure S1 D).

### Phylogenetic analysis of HA sequences of global H4 subtype viruses

**Figure 2.**
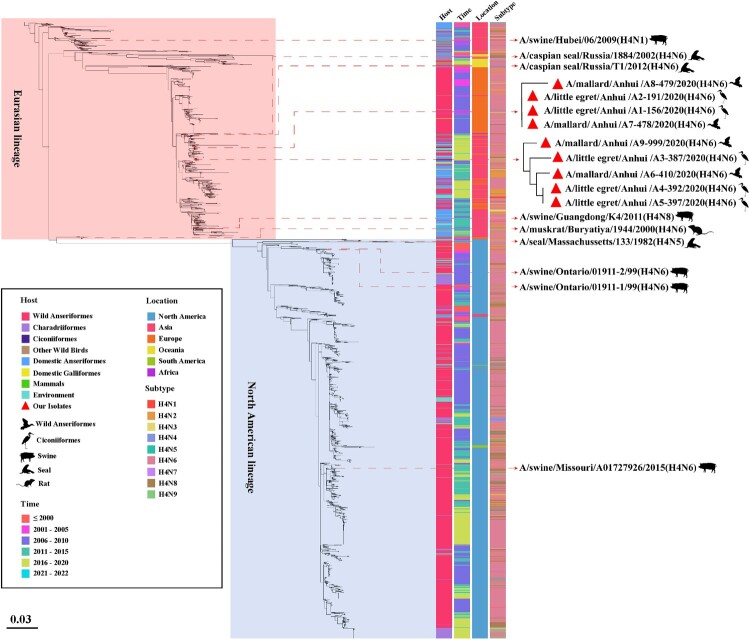
Maximum likelihood phylogenetic tree of 2498 HA genes of global H4 viruses. The phylogenetic tree is constructed using ML method by IQ-TREE v.1.6.12. Nine H4N6 AIVs isolated in this study are marked with red triangles. The tree is further visualized and edited by the Chiplot online tool.

The information about host, collection date, location and subtype of 2498 HA sequences of global H4 viruses was mapped onto the phylogenetic tree (Figure 2, Table S2). As shown in Figure 2, the HA genes of the H4 subtype viruses can be divided into two major lineages, Eurasian and North American lineage, and nine HA genes of H4N6 isolates in this study belong to the Eurasian lineage. It is worth noting that a total of nine viruses were isolated from mammals, such as pigs, seals and mice. These cases indicate that H4 subtype viruses can cross the species barrier to infect mammals, posing a potential threat to public health security.

### Bayesian phylodynamic analysis of H4 viruses belonging to the Eurasian lineage

**Figure 3.**
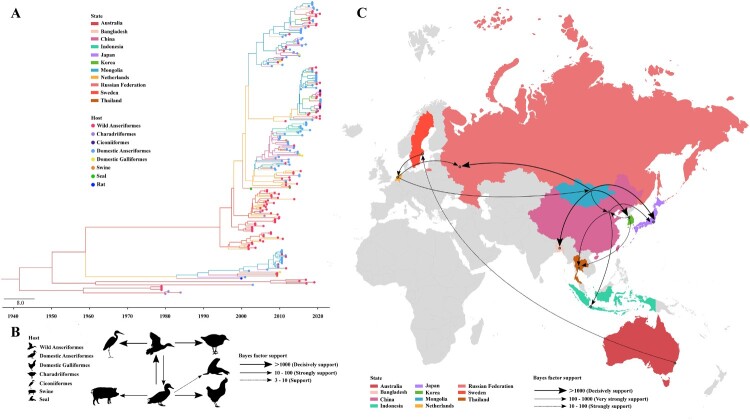
Bayesian phylodynamic analysis of HA genes of H4 viruses in Eurasian lineage. (A) Maximum clade credibility tree of HA sequences of H4 viruses in Eurasian lineage. The HA sequences from different states are indicated by different branch colours. Different coloured dots at the end of the branches represent different hosts. (B) Transmission pattern between different hosts of H4 viruses in Eurasian lineage. Different hosts (wild Anseriformes, domestic Anseriformes, domestic Galliformes, Charadriiformes, Ciconiiformes, swine and seal) are represented by silhouettes. The bold arrows represent decisively supported diffusions (BF > 1000); the solid arrows represent strongly supported diffusions (10 < BF < 100); the dashed arrow represents supported diffusion (3 < BF < 10). (C) Spatial diffusion of H4 viruses in Eurasian lineage. The bold arrows represent decisively supported diffusions (BF > 1000); the solid arrows represent very strongly supported diffusions (100 < BF < 1000); the dashed arrows represent strongly supported diffusion (10 < BF < 100).

To estimate the transmission patterns of Eurasian lineage H4 viruses between regions and species, a Bayesian phylodynamic analysis of HA gene segments of H4 subtype viruses belonging to the Eurasian lineage was performed. The available sequences (n = 229) were divided into 11 states (Mongolia (n = 44), China (n = 40), Netherlands (n = 29), Sweden (n = 28), Russian Federation (n = 23), Japan (n = 13), Australia (n = 12), Bangladesh (n = 10), Indonesia (n = 10), Korea (n = 10) and Thailand (n = 10)) and eight host categories (wild Anseriformes (n = 109), domestic Anseriformes (n = 100), Charadriiformes (n = 5), Ciconiiformes (n = 5), domestic Galliformes (n = 5), swine (n = 2), seal (n = 2) and rat (n = 1)) (Figure 3A).

The transmission pattern of H4 viruses of Eurasian lineage between different host species was shown in Figure 3B. It is speculated that domestic Anseriformes and wild Anseriformes were two intermediate hosts for H4 viruses to spread to other hosts, and there was mutual transmission between two intermediate hosts. The domestic Anseriformes were responsible for the transmission of H4 viruses to domestic Galliformes (BF > 1000), swine (10 < BF < 100) and seal (3 < BF < 10). In addition, wild Anseriformes contributed to the spillover of the viruses to Charadriiformes and Ciconiiformes (both BF > 1000) (Table S3).

Furthermore, we evaluated the migration of H4 viruses of Eurasian lineage, and identified 14 transmission routes (Figure 3C). There were four routes of decisive support with BF larger than 1000, and the four transmission routes were identified from Mongolia to the Russian Federation, Bangladesh, Korea and Japan, respectively (Table S4). These data indicate that Mongolia has acted as the epidemic source for the spread of Eurasian lineage H4 viruses. It is worth noting that the H4 viruses from Mongolia and Korea were responsible for the transmission of the viruses to China, with the BF of 335.0507 and 15.9397, respectively.

### The H4N6 isolates from wild birds in the study formed two genotypes and underwent complex reassortment

According to the ML trees, the HA (Figure S2 A) and NA (Figure S2 B) genes of nine H4N6 isolates belonged to the Eurasian lineage, with nucleotide identities of 98.6% – 100% and 99.6% – 100%, respectively. While the internal genes of nine H4N6 isolates exhibited relative diversity. The PB2 and PA genes fell into the Eurasian lineage, and formed two groups (Figure S3 A and S3 C), sharing nucleotide identities of 93.5% – 100% and 93.7% – 100%, respectively. The remaining PB1, NP, M and NS genes also clustered into the Eurasian lineage (Figure S3 B, S3 D–S3 F), and their nucleotide identities were 99.6% – 100%, 97.3% – 100%, 98.2% – 100% and 99.6% – 100%, respectively. Based on the above phylogenetic analysis, our nine H4N6 viruses isolated from wild birds can be divided into two genotypes (Figure S4), and LG/AH/A1-156/2020(H4N6) and ML/AH/A9-999/2020(H4N6) are selected as two representative strains for further research.

**Figure 4.**
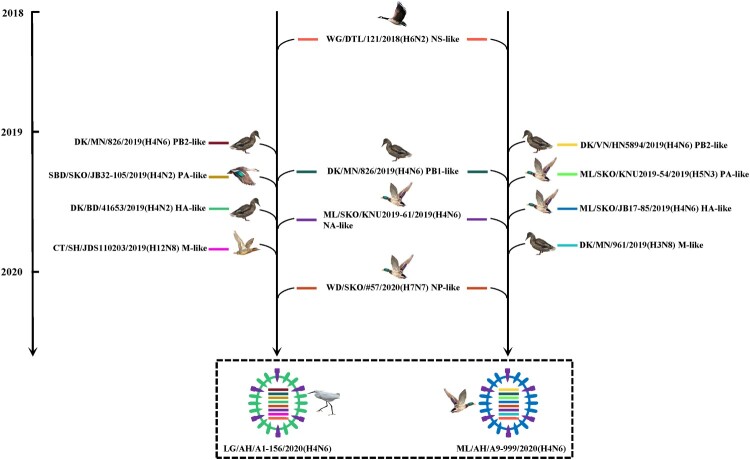
Schematic diagram of gene recombination of two representative H4N6 viruses. Eight gene segments of two H4N6 isolates are represented by horizontal bars in each oval (PB2, PB1, PA, HA, NP, NA, M and NS, from top to bottom).

To trace the origin of nine H4N6 isolates from wild birds, we investigated the formation of two representative LG/AH/A1-156/2020(H4N6) and ML/AH/A9-999/2020(H4N6) by blasting the influenza viruses with highest nucleotide homology in the public source (Table S5). As shown in Figure 4, two representative H4N6 viruses shared the same gene contributors of PB1, NP, NA and NS segments, with DK/MN/826/2019(H4N6)-like, WD/SKO/#57/2020(H7N7)-like, ML/SKO/KNU2019-61/2019(H4N6)-like and WG/DTL/121/2018(H6N2)-like, respectively. In addition, the PB2 gene of LG/AH/A1-156/2020(H4N6) was also the descendant of DK/MN/826/2019(H4N6)-like. The remaining PA, HA and M genes of LG/AH/A1-156/2020(H4N6) were acquired from SBD/SKO/JB32-105/2019(H4N2)-like, DK/BD/41653/2019(H4N2)-like and CT/SH/JDS110203/2019(H12N8)-like, respectively. For ML/AH/A9-999/2020(H4N6), the PB2 and M genes were introduced from an H4N6 and an H3N8 virus isolated from ducks in Vietnam (DK/VN/HN5894/2019(H4N6)-like) and in South Korea (DK/MN/961/2019(H3N8)-like), respectively; its PA and HA genes derived from an H5N3 and an H4N6 LPAIV in mallards in South Korea, with ML/SKO/KNU2019-54/2019(H5N3)-like and ML/SKO/JB17-85/2019(H4N6)-like being their gene donors, respectively. These data suggest that the emergence of nine H4N6 viruses from wild birds represented by LG/AH/A1-156/2020(H4N6) and ML/AH/A9-999/2020(H4N6) in this study was caused by complex genetic recombination, and they were reassorted by various subtype LPAIVs from wild birds and poultry.

### The H4N6 isolates from wild birds possessed amino acid mutations associated with enhanced pathogenicity in mammals

The HA genes of nine H4N6 isolates from wild birds encoded PEKASR↓GLF amino acid sequences at the cleavage site, suggesting that they accorded with the molecular characteristics of LPAIVs [33]. It should be noted that nine H4N6 viruses had amino acid mutations associated with enhanced pathogenicity in mammals, including D3V and D622G in PB1 protein [34,35], N66S in PB1-F2 protein [36], N30D, I43M and T215A in M1 protein [37,38] and P42S and I106M in NS1 protein [39,40] (Table 1).

**Table 1.** Molecular markers of H4N6 viruses from wild birds in the study.

Virus name	Increased virus binding to human-type receptor				Increased virulence in mice and increased polymerase activity					Increased virulence in mice								Reduced susceptibility to zanamivir	
	HA (H3 numbering)^a^				PB2			PB1		NA (N2 numbering)^a^	PB1-F2	M1			NS1			NA (N2 numbering)	
	E 190 G	G 225 D	Q 226 L	G 228 A/S	E 158 K	E 627 K	D 701 N	D 3 V	D 622 G	Stalk deletion	N 66 S	N 30 D	I 43 M	T 215 A	80–84 deletion	*P* 42 S	I 106 M	E 119 A/D/G	R 292 K
LG/AH/A1-156/2020(H4N6)	–^b^	–	–	–	–	–	–	V	G	–	S	D	M	A	–	S	M	–	–
LG/AH/A2-191/2020(H4N6)	–	–	–	–	–	–	–	V	G	–	S	D	M	A	–	S	M	–	–
LG/AH/A3-387/2020(H4N6)	–	–	–	–	–	–	–	V	G	–	S	D	M	A	–	S	M	–	–
LG/AH/A4-392/2020(H4N6)	–	–	–	–	–	–	–	V	G	–	S	D	M	A	–	S	M	–	–
LG/AH/A5-397/2020(H4N6)	–	–	–	–	–	–	–	V	G	–	S	D	M	A	–	S	M	–	–
ML/AH/A6-410/2020(H4N6)	–	–	–	–	–	–	–	V	G	–	S	D	M	A	–	S	M	–	–
ML/AH/A7-478/2020(H4N6)	–	–	–	–	–	–	–	V	G	–	S	D	M	A	–	S	M	–	–
ML/AH/A8-479/2020(H4N6)	–	–	–	–	–	–	–	V	G	–	S	D	M	A	–	S	M	–	–
ML/AH/A9-999/2020(H4N6)	–	–	–	–	–	–	–	V	G	–	S	D	M	A	–	S	M	–	–

^a^ The mutations/motifs are numbered according to alignments with A/Aichi/2/1968(H3N2).

^b^ The “–” indicates the mutation was not detected in the H4N6 viruses.

### The H4N6 isolates from wild birds exhibited dual receptor binding specificity

**Figure 5.**
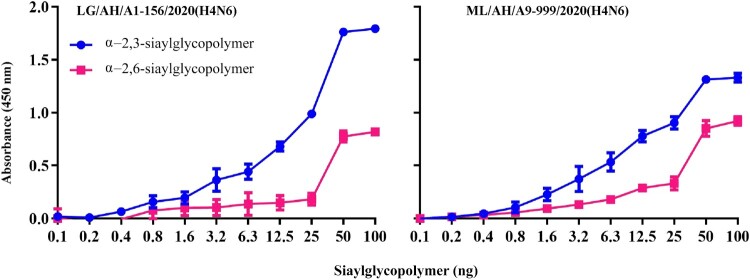
Receptor binding specificity of two representative H4N6 viruses. The binding affinities of two tested viruses for avian-type and human-type receptors were analyzed by two different glycans (α−2,6-siaylglycopolymer, red; α−2,3-siaylglycopolymer, blue). The data shown are the means of three repeats and the error bars indicate the standard deviations.

To further investigate the receptor binding specificity of the H4N6 viruses from wild birds, we assessed the receptor binding affinities of two representative viruses with α−2,3-siaylglycopolymer and α−2,6-siaylglycopolymer by solid-phase binding experiments. As shown in Figure 5, two representative LG/AH/A1-156/2020(H4N6) and ML/AH/A9-999/2020(H4N6) exhibited dual receptor binding properties. Although two tested viruses had a higher affinity for avian-type receptors, they acquired the ability to bind to human-type receptors.

### The H4N6 isolates from wild birds could effectively replicate in MDCK and 293 T cells

**Figure 6.**
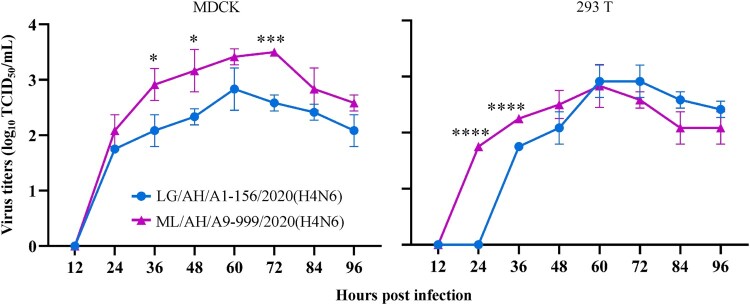
Growth curves of two representative H4N6 viruses in MDCK and 293 T cells. The statistical significance was based on Student’s t-test using GraphPad Prism 8 software (*, *P* < 0.05; **, *P* < 0.01; ***, *P* < 0.001; ****, *P* < 0.0001). Three biological replicates were conducted for each sample.

To confirm the replication ability of H4N6 isolates from wild birds in mammalian cells, two representative LG/AH/A1-156/2020(H4N6) and ML/AH/A9-999/2020(H4N6) were used to infect MDCK and 293 T cells at an MOI of 0.01. As shown in Figure 6, both the tested viruses grew well in MDCK and 293 T cells. It is worth noting that ML/AH/A9-999/2020(H4N6) exhibited significantly higher titers than LG/AH/A1-156/2020(H4N6) at hour 36, 48 and 72 p.i. (*, *P* < 0.05; ***, *P* < 0.001) in MDCK cells, and at hour 24 and 36 p.i. (****, *P* < 0.0001) in 293 T cells.

### The H4N6 isolates from wild birds could infect mice directly

**Figure 7.**
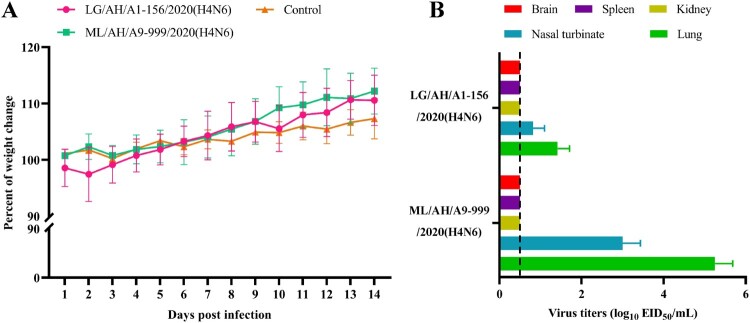
Pathogenicity and replication of two representative H4N6 viruses in mice. (A) Body weight changes in inoculation groups and control group of five mice after inoculation with 10^6.0^ EID_50_ of the viruses and PBS, respectively. (B) Virus titers in organs of mice on day 3 p.i. were tested. The dashed line indicates the lower limit of detection. The error bars represent the standard deviation.

To assess the pathogenicity of the H4N6 isolates from wild birds in mammals, BALB/c mice were infected with two representative LG/AH/A1-156/2020(H4N6) and ML/AH/A9-999/2020(H4N6) as described previously at a dose of 10^6.0^ EID_50_, respectively [41]. As shown in Figure 7A, LG/AH/A1-156/2020(H4N6) caused slight body weight loss (about 2.5%) on day 2 p.i. in mice, however, the mice gradually recovered beginning on day 3 p.i.. While the mice infected with ML/AH/A9-999/2020(H4N6) gradually gained weight during the observation period. Besides, on day 3 p.i., we found two representative LG/AH/A1-156/2020(H4N6) and ML/AH/A9-999/2020(H4N6) viruses could be detected in lungs of mice, with titers ranging from 1.25 – 1.75 log_10_ EID_50_ and from 4.75 – 5.50 log_10_ EID_50_, respectively, and in nasal turbinates of mice, with titers ranging from 0.50 – 0.98 log_10_ EID_50_ and from 2.50 – 3.25 log_10_ EID_50_, respectively (Figure 7B). While no virus was observed in brains, spleens or kidneys. The results show that the H4N6 isolates from wild birds could infect mice directly without adaptation, and ML/AH/A9-999/2020(H4N6) exhibited greater replication ability than LG/AH/A1-156/2020(H4N6) in mice.

## Discussion

Wild birds are thought to be the natural reservoirs for AIVs [42], and H4 subtype viruses can spread worldwide by the migration of wild birds [15]. To better understand the global prevalence of H4 viruses, we obtained a total of 2498 HA sequences of H4 viruses in the public database for time and geographical distribution statistics. The statistical results showed that the highest number of isolates was in North America (n = 1597; 63.9%), followed by Asia (n  =  488; 19.5%) and Europe (n = 351; 14.0%), while the number of H4 viruses isolated from Oceania (n  =  45; < 2%), South America (n  =  12; < 2%) and Africa (n  =  5; < 2%) was relatively small. From the perspective of hosts, the most H4 viruses were isolated from wild birds (n  =  2024; 81.0%), followed by poultry (n  =  342; 13.6%) and environment (n  =  123; 4.9%). It should be noted that there were nine H4 viruses originating from mammals, indicating that H4 viruses possessed the ability to infect mammals, posing a potential threat to global public health security. In addition, we found the number of H4 isolates began to increase significantly in 2006 and reached its highest level of 285 in 2009. In terms of subtypes, global H4 viruses have multiple subtype combinations (H4N1 – H4N9), with the highest number occurred in H4N6 subtype.

Bayesian phylodynamic analysis of HA genes of Eurasian lineage H4 viruses revealed that wild Anseriformes and domestic Anseriformes served as the two intermediate hosts for H4 viruses to spread to other hosts, and domestic Anseriformes were responsible for the transmission of viruses to mammals (swine and seal). It is reported that live poultry markets are important sites of human infection with AIVs [43], due to the frequent contact between humans and poultry, whether H4 viruses will be transmitted to humans through poultry in the future deserves continued public attention. Furthermore, three migration routes for migratory birds, the East Africa-West Asia flyway, Central Asia flyway and East Asia-Australia flyway, are through Mongolia, meaning that wild migratory birds from Mongolia might carry AIVs and spread them throughout the world. Also, this study confirms that Mongolia was the important transmission centre of the H4 subtype viruses in the Eurasian lineage.

Gene recombination plays a crucial role in the evolution of influenza viruses and the emergence of pandemic strains [44]. It is reported that clade 2.3.4.4b H5N8 virus has caused outbreaks in wild birds and poultry in Europe, Africa and Asia from January 2020 to October 2021, and the virus reassorted with other subtypes of AIVs to form different H5 viruses bearing clade 2.3.4.4b HA gene, including H5N1, H5N2, H5N3, H5N4, H5N5 and H5N6. Among them, the H5N1 virus has become the dominant strain causing avian influenza outbreaks worldwide [45,46]. In this study, the emergence of H4N6 AIVs from wild birds, represented by LG/AH/A1-156/2020(H4N6) and ML/AH/A9-999/2020(H4N6), was the result of complex genetic recombination between different subtypes of LPAIVs from Asian countries. Therefore, the continued recombination of H4 AIVs from wild birds deserves close public attention.

Binding to human-type receptors is considered to be a prerequisite for the effective transmission of influenza viruses from human to human [47]. Previous study has shown that amino acid mutations at site 190, 225, 226 and 228 in the HA protein (H3 numbering) of H4 viruses are important for changes in receptor binding properties [48]. We further analyzed and visualized the sequence mutants at the above four sites in HA proteins of 2489 H4 viruses from GISAID EpiFlu database through WebLogo3 (https://weblogo.threeplusone.com/create.cgi) (Figure S5, Table S6), and the results showed that one-third of H4 viruses isolated from mammals harboured the Q226L and G228S mutations in their HA proteins, leaving the hidden danger for H4 viruses to infect humans. In this study, it is worth noting that both two representative LG/AH/A1-156/2020(H4N6) and ML/AH/A9-999/2020(H4N6) possessed dual receptor binding specificities, and they acquired the ability to bind to human-type receptors, revealing the potential threat of H4N6 AIVs from wild birds to mammals. The confirmed mutations associated with human-type receptor binding preference were not found, and whether existing other mutations that cause the H4N6 viruses to exhibit dual receptor binding characteristics needs to be further investigated. The acquisition of mammalian adaptive mutations contributes to the efficient replication and spread of AIVs in humans [49]. Notably, nine H4N6 isolates in this study acquired several virulence-enhanced mutations. We then explored the ability of H4N6 AIVs from wild birds to infect mammals both in vitro and in vivo. The results indicated that two representative LG/AH/A1-156/2020(H4N6) and ML/AH/A9-999/2020(H4N6) could effectively replicate in mammalian MDCK and 293 T cells. Moreover, both two tested viruses were able to infect BALB/c mice without adaptation, and could replicate in lungs and nasal turbinates of mice. Here, we only investigated the biological characteristics of H4N6 viruses from wild birds, while those of H4N6 viruses isolated from domestic poultry need to be further explored.

While the biological characteristics of H4N6 AIVs from wild birds have been thoroughly investigated, they can bind to human-type receptor and can effectively replicate in mammalian cells and mice, and we emphasize that the monitoring of H4N6 virus circulating in wild birds should continue to be strengthened.

## Supplementary Material

Table S4 Bayes factor for location transmission of H4 viruses in the Eurasian lineage.docx

Figure S2 ML phylogenetic trees of the external genes of nine H4N6 isolates from wild birds.pdf

Table S1 H4N6 AIVs isolated from wild birds in Anhui Province China 2020.docx

Table S6 Mutations in the HA of H4 viruses in GISAID EpiFlu database that may increase the affinity to human type receptor.docx

Figure S5 The key receptor binding site statistics of H4 viruses in GISAID EpiFlu database.pdf

Table S2 Global H4 subtype viruses information.xlsx

Figure S1 Spatial and temporal distribution of H4 subtype viruses in China.pdf

Figure S3 ML phylogenetic trees of the internal genes of nine H4N6 isolates from wild birds.pdf

Table S3 Bayes factor for host transmission of H4 viruses in the Eurasian lineage.docx

Figure S4 The genotypes of nine H4N6 isolates from wild birds.pdf

Table S5 The highest nucleotide homology of the whole genomes of two representative H4N6 viruses.docx

## Data Availability

Genomes sequences of nine H4N6 AIVs isolated in this study were uploaded to GISAID EpiFlu database (Accession ID: EPI3513363 – EPI3513434).
