# Supplementary material for: Epidemiology and biological characteristics of influenza A (H4N6) viruses from wild birds
Source: Emerg Microbes Infect. 2024 Oct 17;13(1):2418909. doi: 10.1080/22221751.2024.2418909 (PMC11523250; doi:10.1080/22221751.2024.2418909)
Supplement: Table S4 Bayes factor for location transmission of H4 viruses in the Eurasian lineage.docx [file TEMI_A_2418909_SM8394.docx]

**Table S4**. Bayes factor for location transmission of H4 viruses in the Eurasian lineage.

| **From** | **To** | **Bayes factor** | **Posterior probability** |
| --- | --- | --- | --- |
| Mongolia | Russian Federation | 257555.3205 | 1 |
| Mongolia | Bangladesh | 257555.3205 | 1 |
| Mongolia | Korea | 4943.8785 | 0.9981 |
| Mongolia | Japan | 2566.3591 | 0.9963 |
| Sweden | Netherlands | 948.2022 | 0.9903 |
| China | Thailand | 857.9339 | 0.9892 |
| China | Indonesia | 708.1631 | 0.9870 |
| Mongolia | China | 335.0507 | 0.9730 |
| Netherlands | Mongolia | 297.3375 | 0.9697 |
| China | Japan | 181.5016 | 0.9513 |
| Netherlands | Russian Federation | 138.9090 | 0.9373 |
| Australia | Sweden | 59.9880 | 0.8659 |
| Korea | Thailand | 20.2570 | 0.6856 |
| Korea | China | 15.9397 | 0.6318 |
