# Supplementary figures and images for: Epidemiology and biological characteristics of influenza A (H4N6) viruses from wild birds
Source: Emerg Microbes Infect. 2024 Oct 17;13(1):2418909. doi: 10.1080/22221751.2024.2418909 (PMC11523250; doi:10.1080/22221751.2024.2418909)

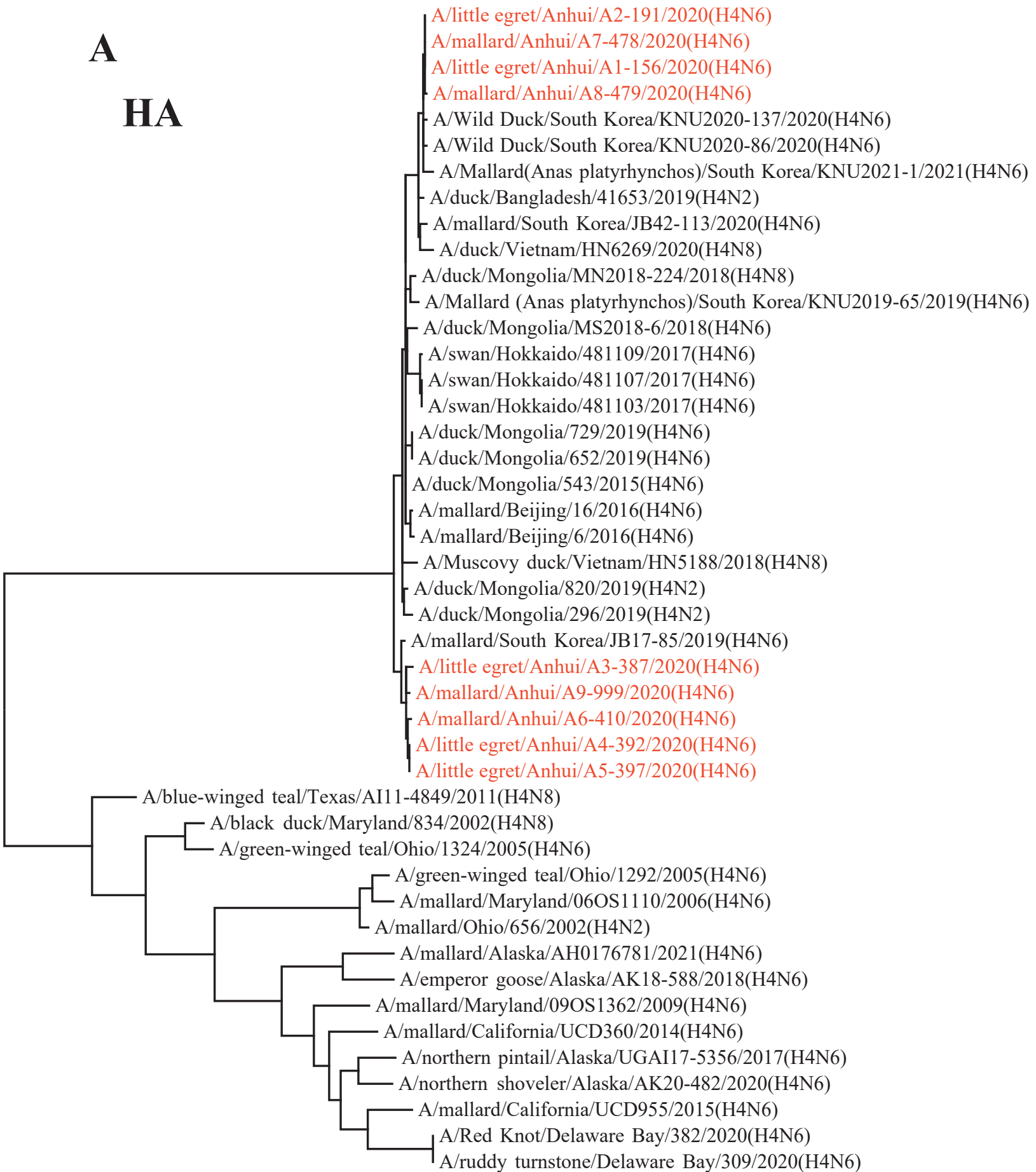

0.02

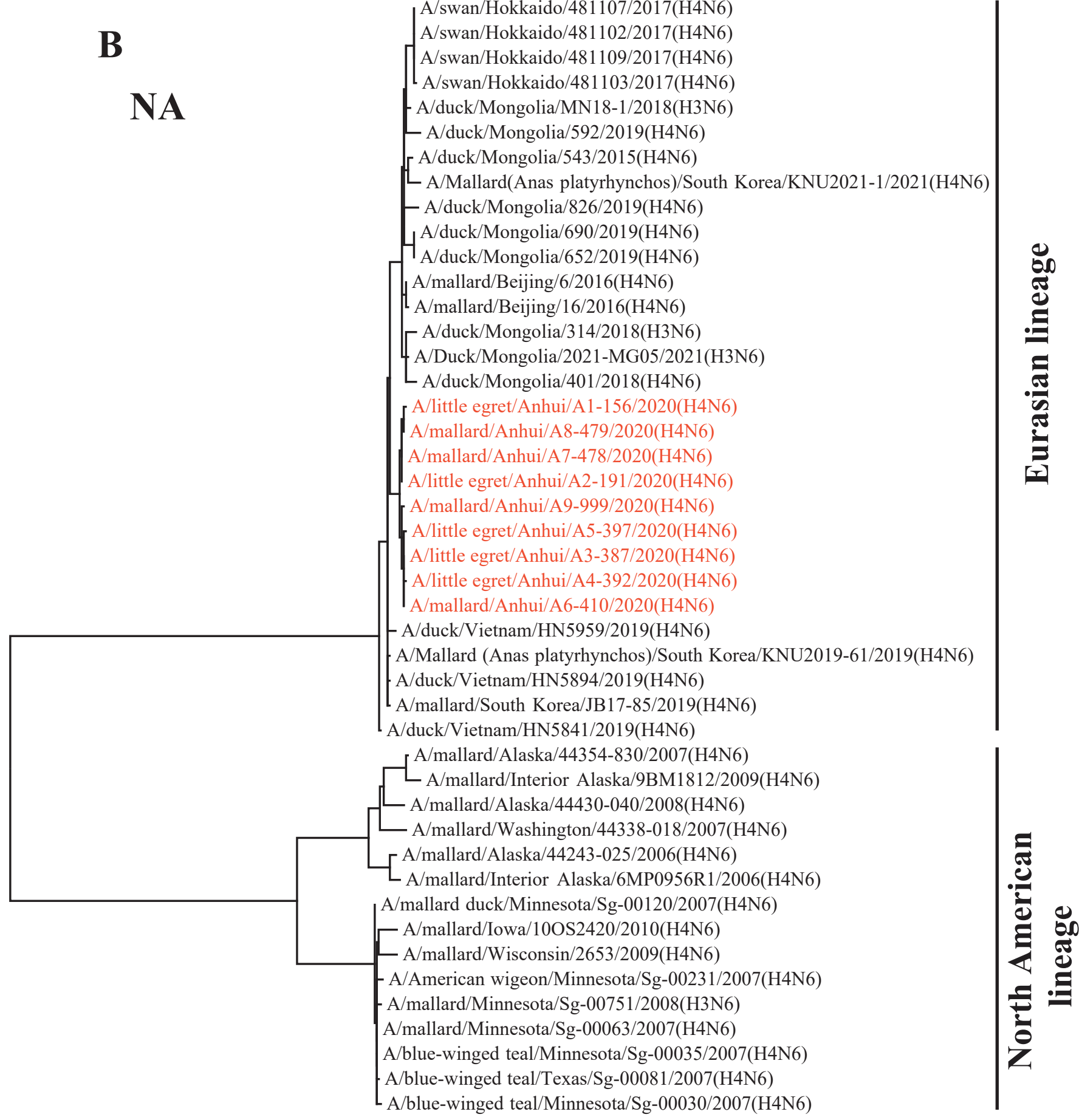

0.02

Supplement: Figure S2 ML phylogenetic trees of the external genes of nine H4N6 isolates from wild birds.pdf [file TEMI_A_2418909_SM8393.pdf]

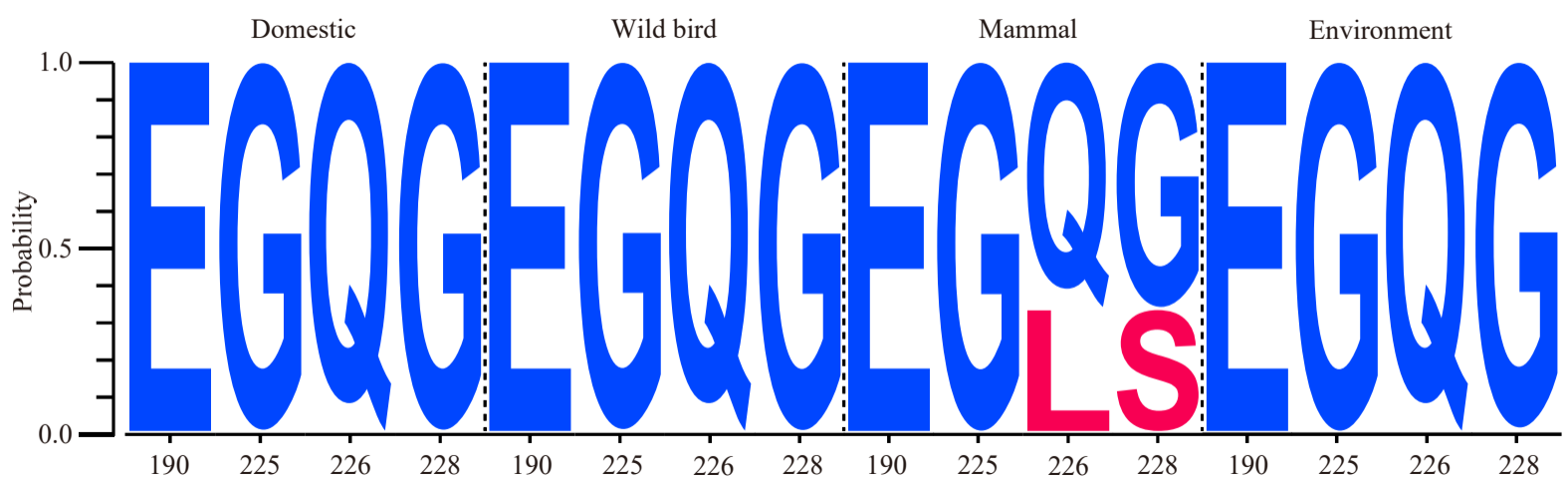

Supplement: Figure S5 The key receptor binding site statistics of H4 viruses in GISAID EpiFlu database.pdf [file TEMI_A_2418909_SM8390.pdf]

**A**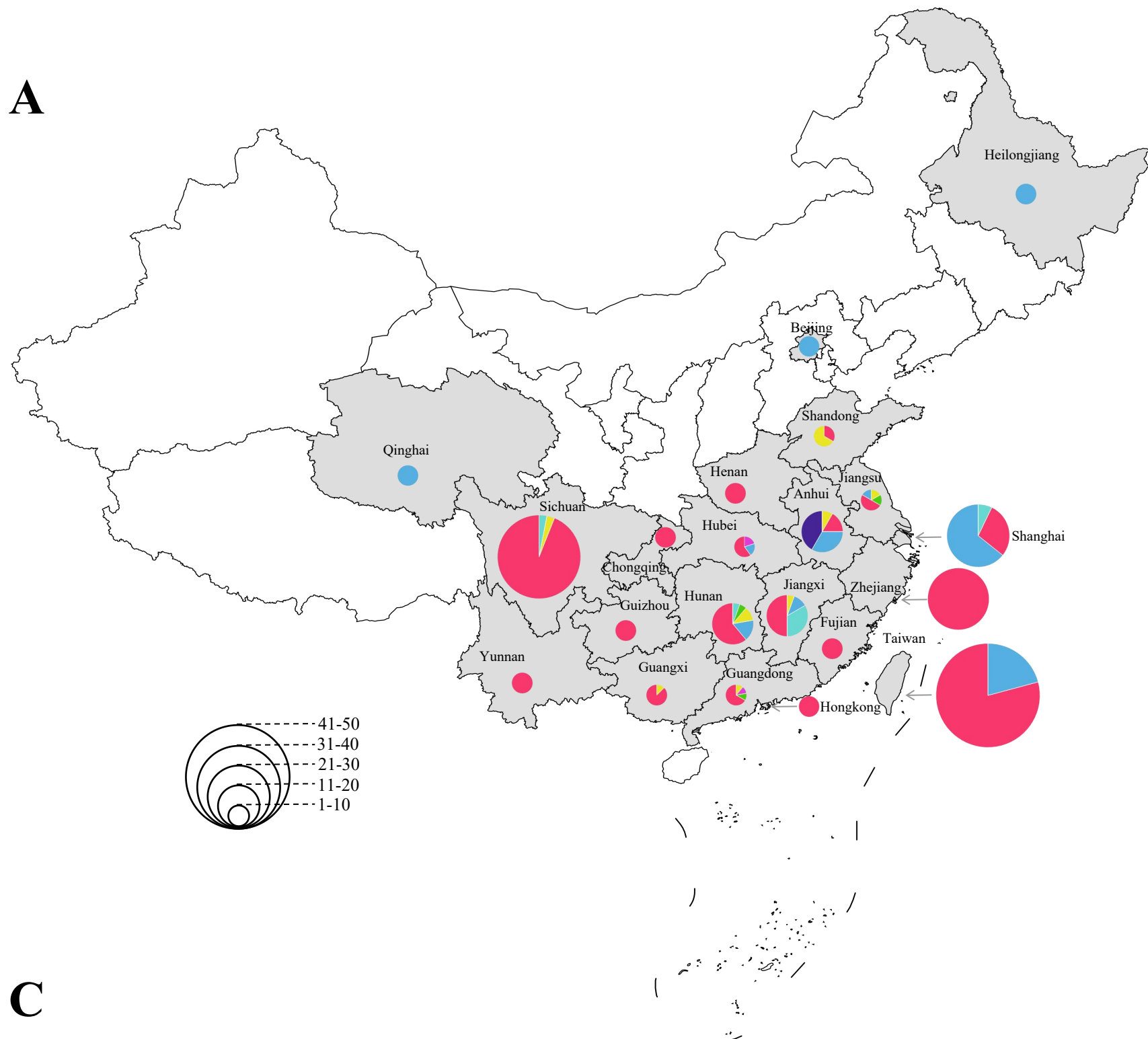**B**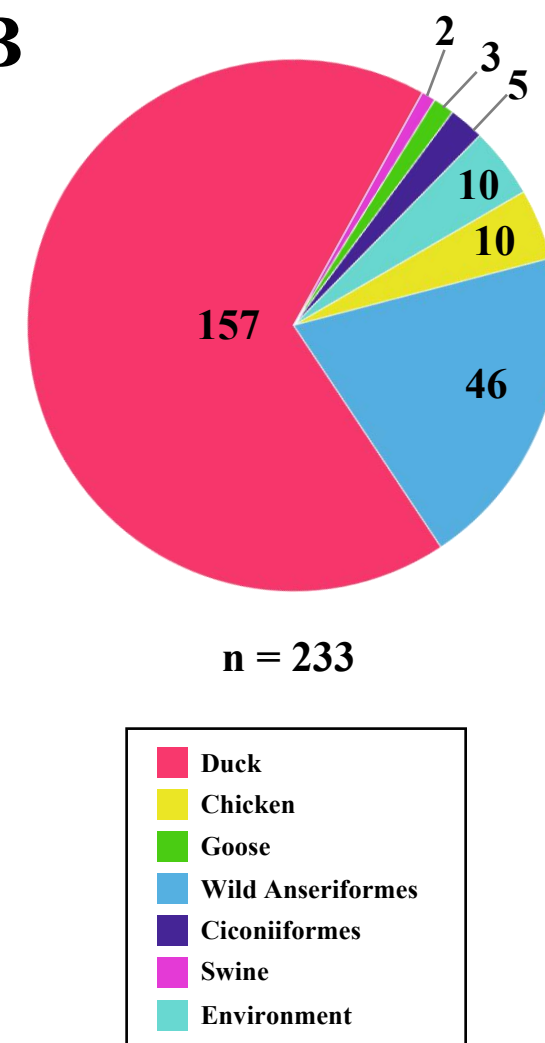**C**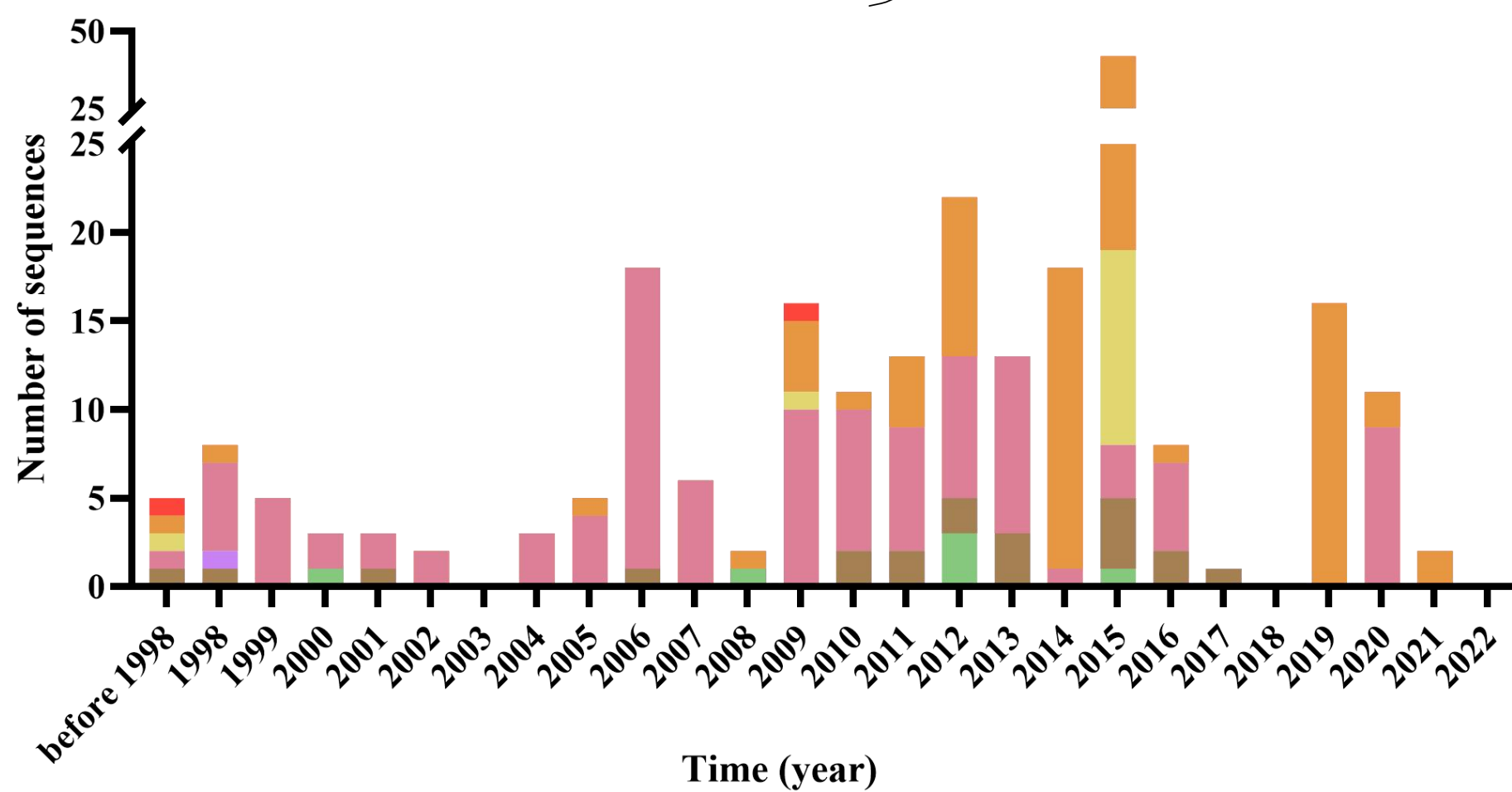**D**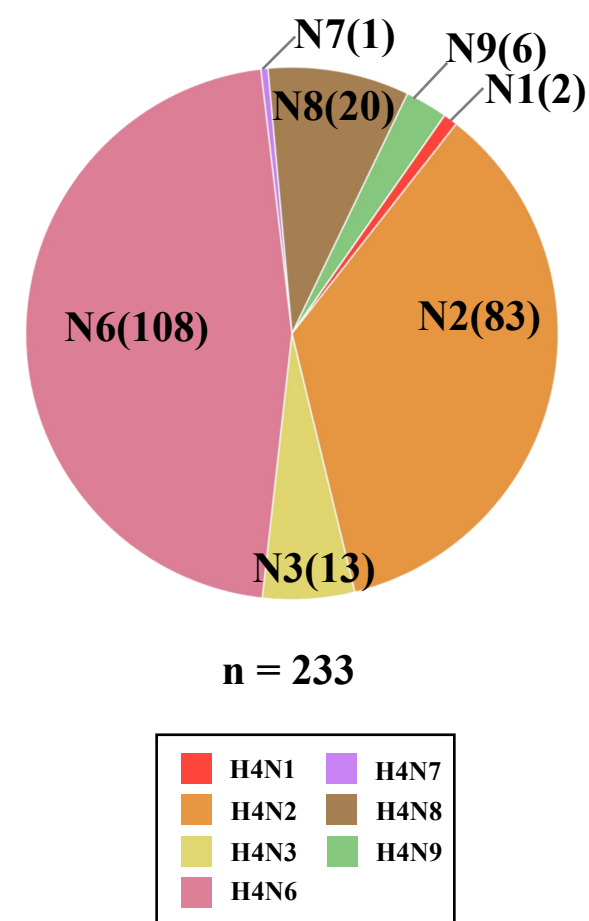

Supplement: Figure S1 Spatial and temporal distribution of H4 subtype viruses in China.pdf [file TEMI_A_2418909_SM8388.pdf]

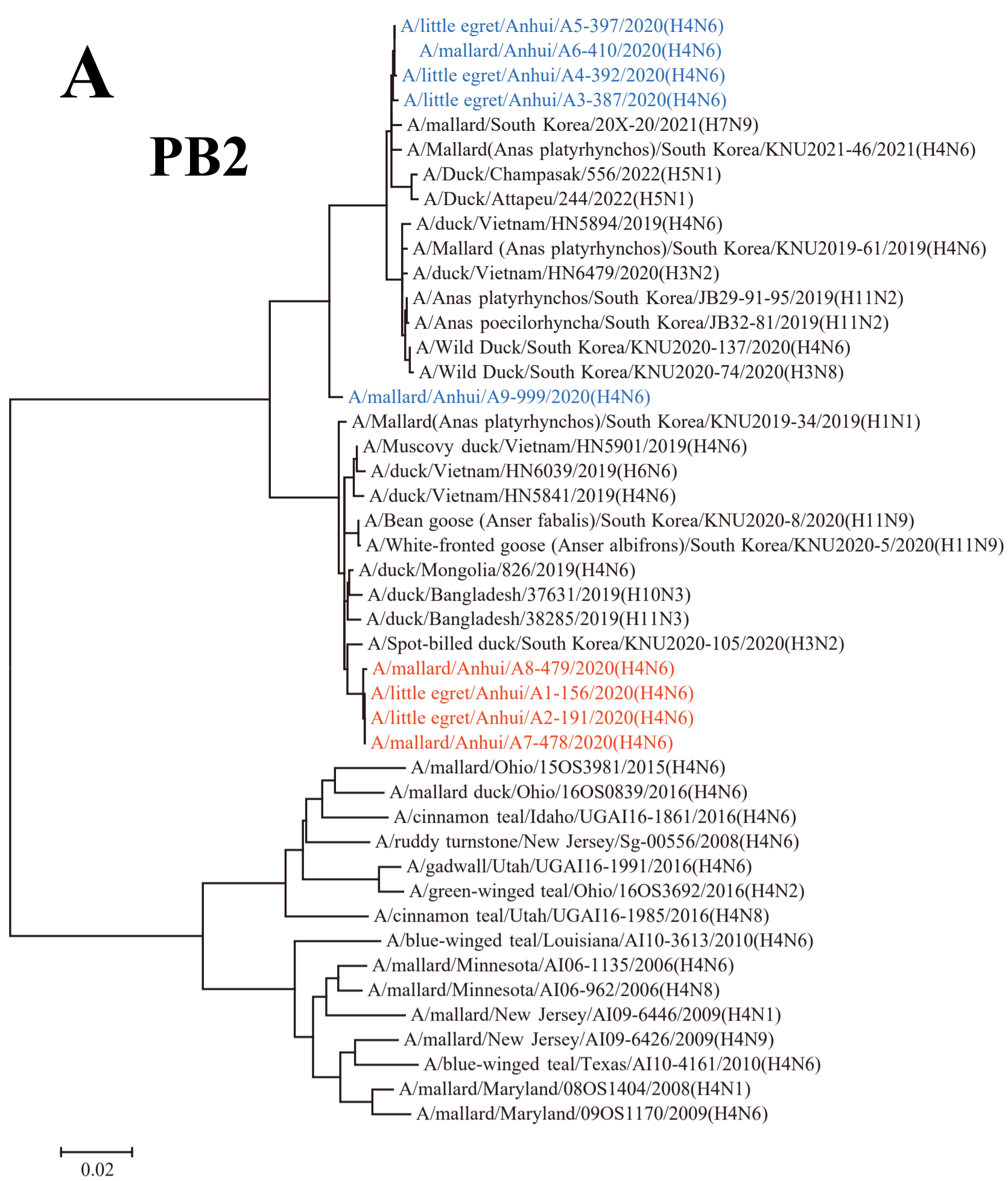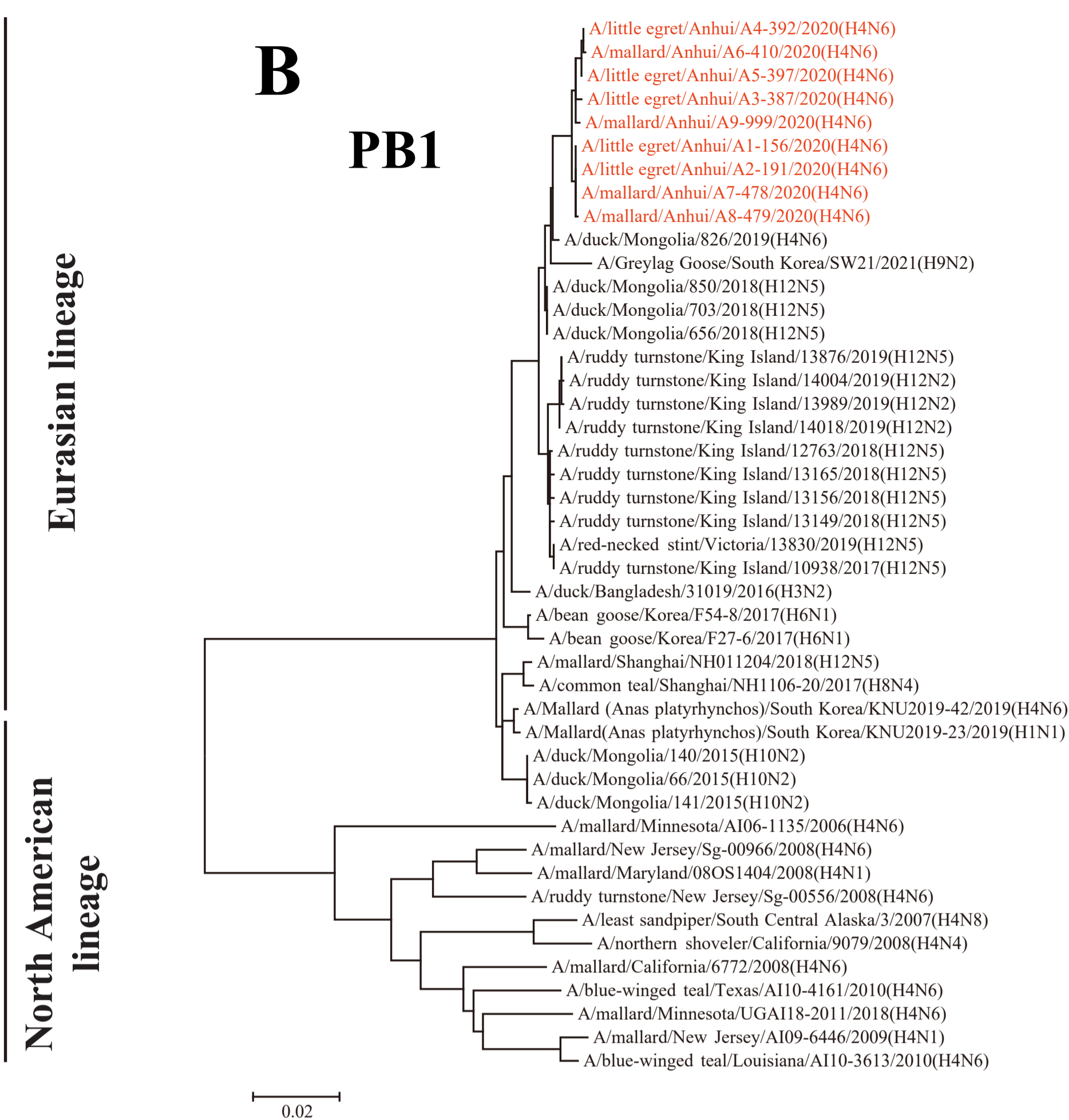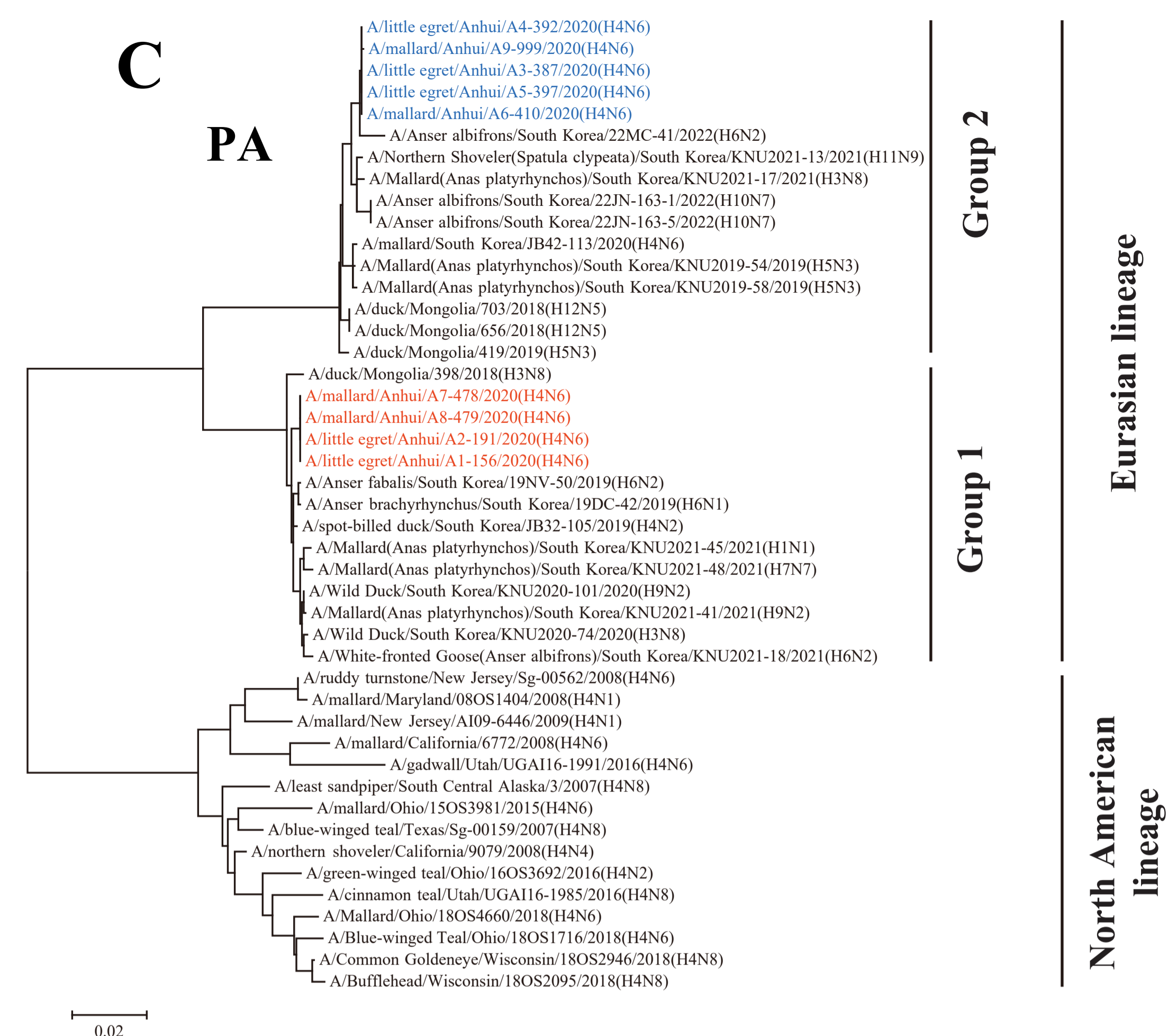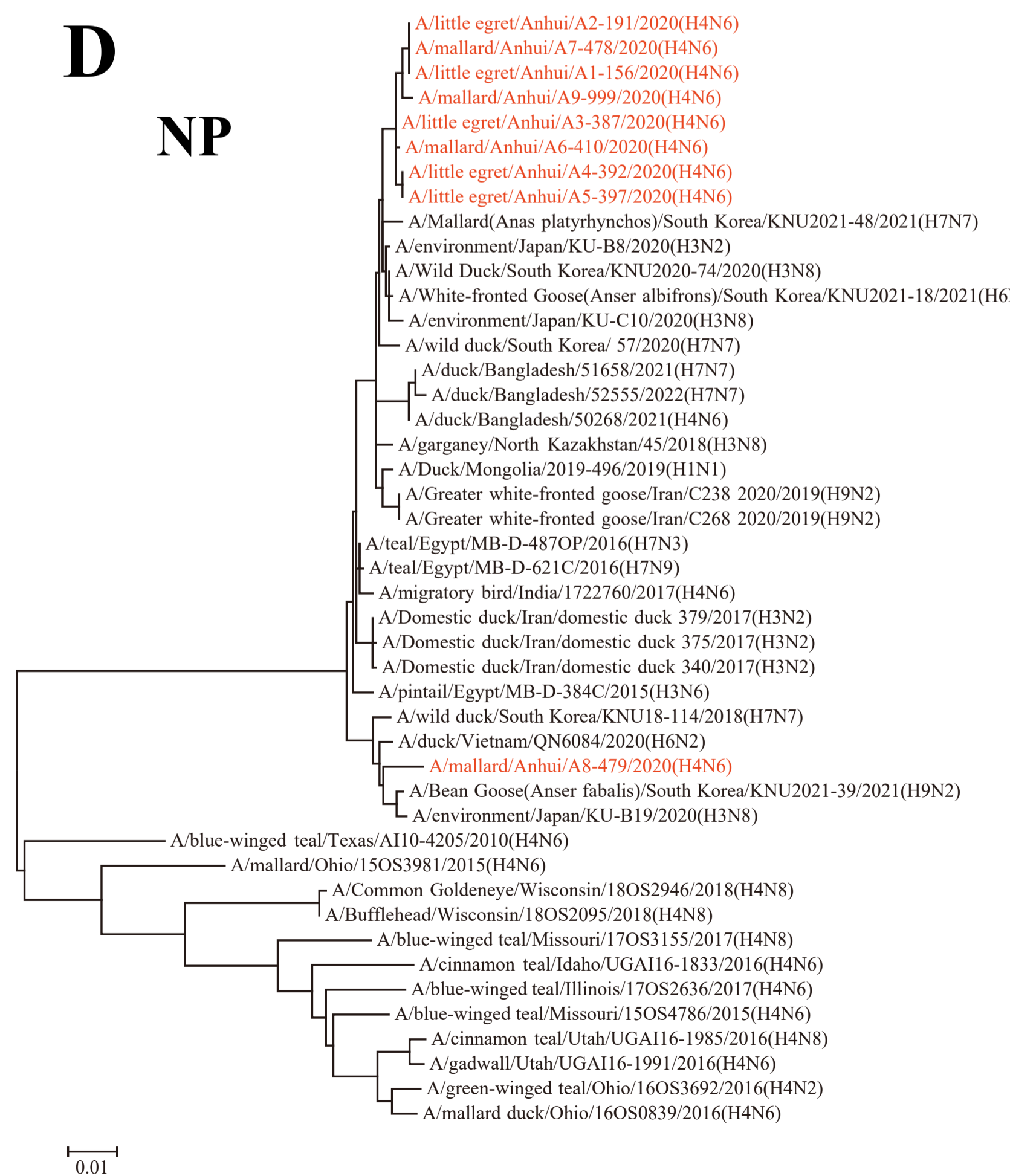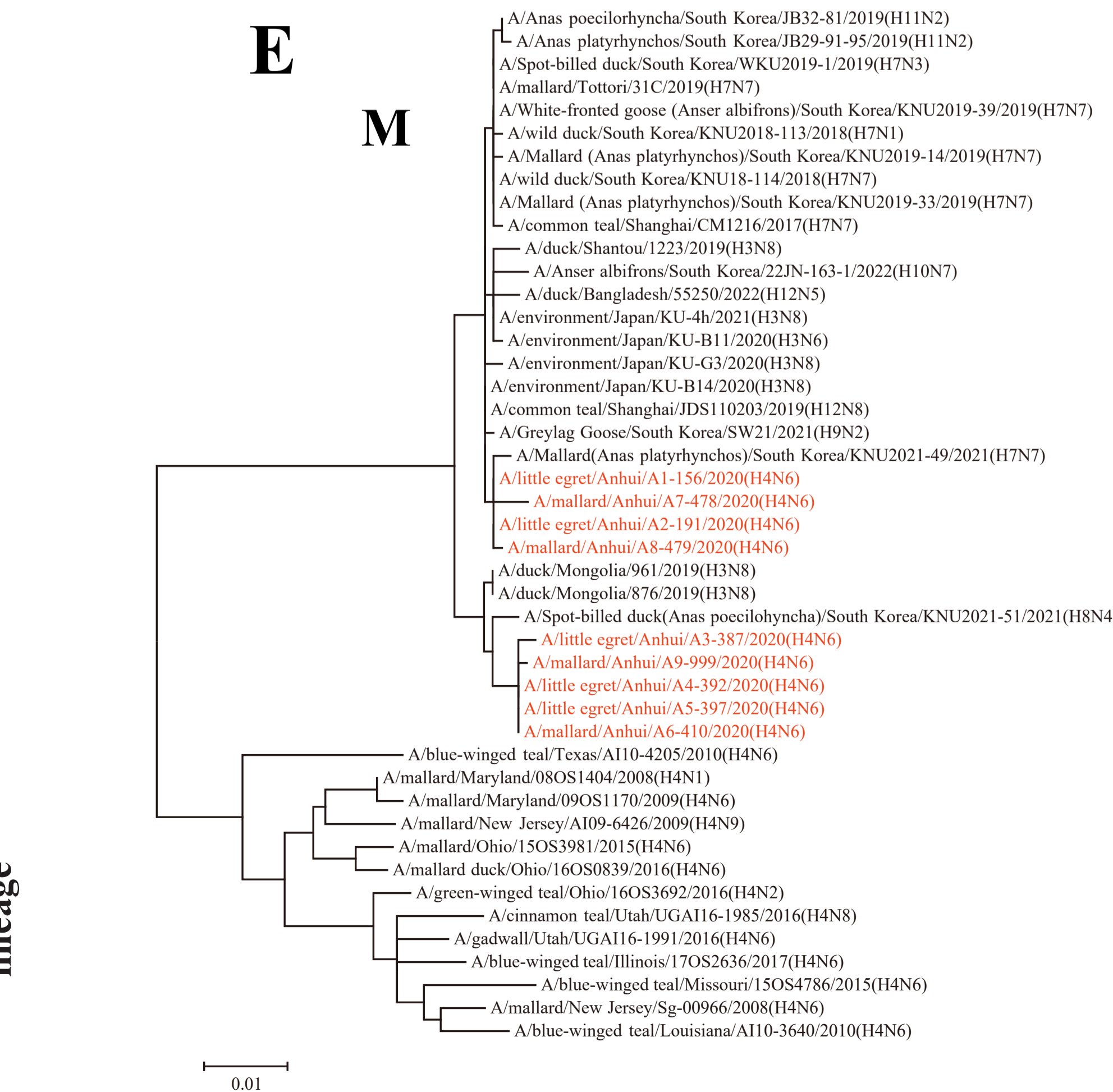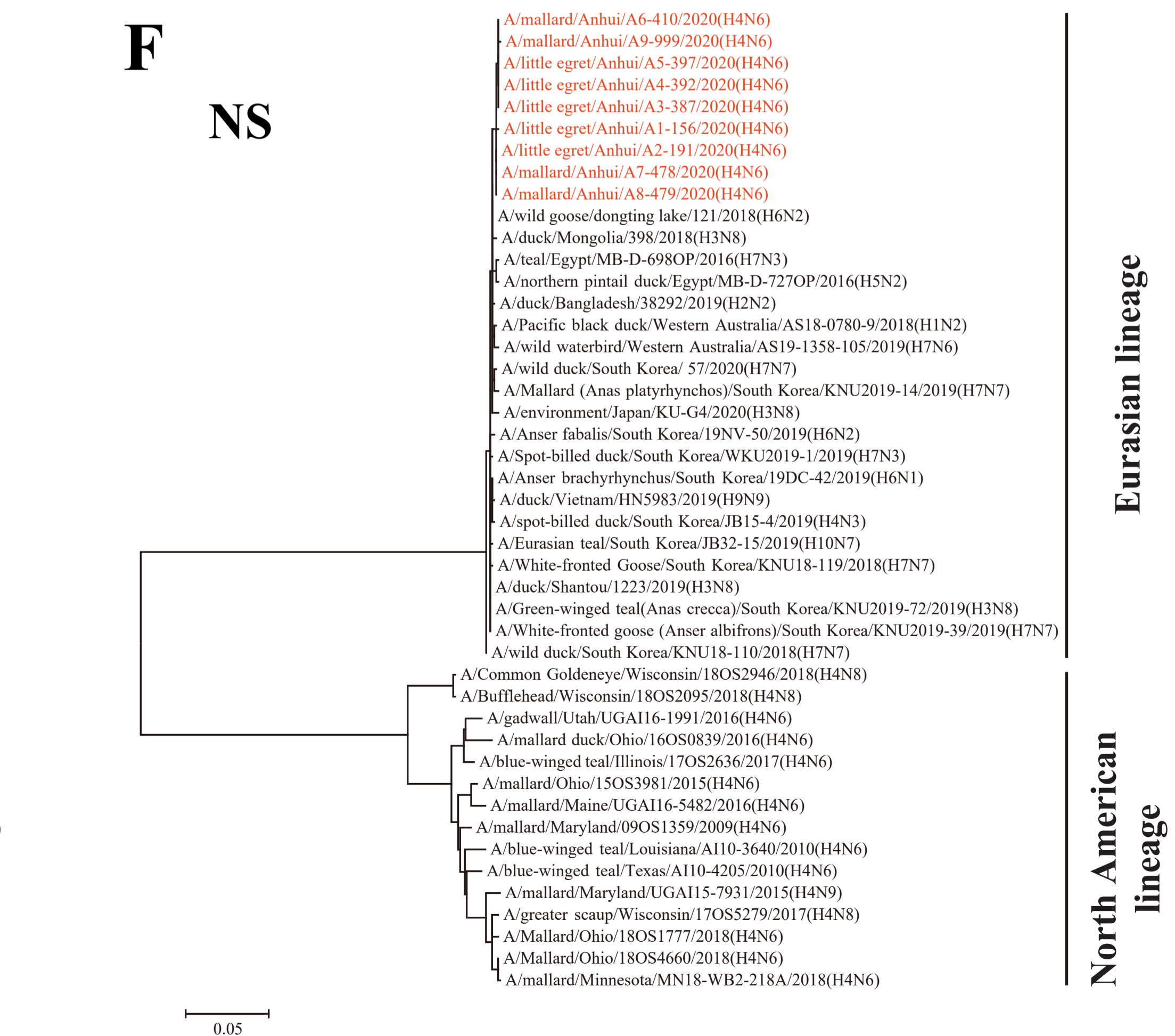

Supplement: Figure S3 ML phylogenetic trees of the internal genes of nine H4N6 isolates from wild birds.pdf [file TEMI_A_2418909_SM8387.pdf]
