# Supplementary material for: Epidemiology and biological characteristics of influenza A (H4N6) viruses from wild birds
Source: Emerg Microbes Infect. 2024 Oct 17;13(1):2418909. doi: 10.1080/22221751.2024.2418909 (PMC11523250; doi:10.1080/22221751.2024.2418909)
Supplement: Table S1 H4N6 AIVs isolated from wild birds in Anhui Province China 2020.docx [file TEMI_A_2418909_SM8392.docx]

**Table S1**. H4N6 AIVs isolated from wild birds in Anhui Province, China, 2020.

| **Sample** | | **AIV isolates** | | | **H4N6 subtype AIVs** | |
| --- | --- | --- | --- | --- | --- | --- |
| **No.** | **Type** | **No.** | **Positive rate (%)** | **Subtype (No.)** | **Name** | **Abbreviation** |
| 1017 | Feces | 17 | 1.67 | H4N6 (9) | A/little egret/Anhui/A1-156/2020 | LG/AH/A1-156/2020 |
|  |  |  |  |  | A/little egret/Anhui/A2-191/2020 | LG/AH/A2-191/2020 |
|  |  |  |  |  | A/little egret/Anhui/A3-387/2020 | LG/AH/A3-387/2020 |
|  |  |  |  |  | A/little egret/Anhui/A4-392/2020 | LG/AH/A4-392/2020 |
|  |  |  |  |  | A/little egret/Anhui/A5-397/2020 | LG/AH/A5-397/2020 |
|  |  |  |  |  | A/mallard/Anhui/A6-410/2020 | ML/AH/A6-410/2020 |
|  |  |  |  |  | A/mallard/Anhui/A7-478/2020 | ML/AH/A7-478/2020 |
|  |  |  |  |  | A/mallard/Anhui/A8-479/2020 | ML/AH/A8-479/2020 |
|  |  |  |  |  | A/mallard/Anhui/A9-999/2020 | ML/AH/A9-999/2020 |
|  |  |  |  | Multiple^a^ (8) |  |  |

a: The eight AIVs are H2N3 (1), H3N8 (1), H5N8 (3), H6N2 (1), H6N8 (1) and H12N2 (1).
