# Supplementary material for: Epidemiology and biological characteristics of influenza A (H4N6) viruses from wild birds
Source: Emerg Microbes Infect. 2024 Oct 17;13(1):2418909. doi: 10.1080/22221751.2024.2418909 (PMC11523250; doi:10.1080/22221751.2024.2418909)
Supplement: Table S6 Mutations in the HA of H4 viruses in GISAID EpiFlu database that may increase the affinity to human type receptor.docx [file TEMI_A_2418909_SM8391.docx]

**Table S6**. Mutations in the HA of H4 viruses in GISAID EpiFlu database that may increase the affinity to human-type receptor.

| **Host type**  **(No. strains)** | **Mutations in the HA that may increase the affinity to human-type receptor**  **(H3 numbering)** | | | |
| --- | --- | --- | --- | --- |
|  | **E190G** | **G225D** | **Q226L** | **G228A/S** |
| Domestic (350) | E (350) | G (349)  E (1) | Q (350) | G (349)  A (1) |
| Wild bird (2007) | E (2007) | G (1998)  S (8)  D (1) | Q (2007) | G (2003)  A (3)  S (1) |
| Mammal (9) | E (9) | G (9) | Q (6)  L (3) | G (6)  S (3) |
| Environment (123) | E (123) | G (123) | Q (123) | G (122)  S (1) |
