# Supplementary material for: Epidemiology and biological characteristics of influenza A (H4N6) viruses from wild birds
Source: Emerg Microbes Infect. 2024 Oct 17;13(1):2418909. doi: 10.1080/22221751.2024.2418909 (PMC11523250; doi:10.1080/22221751.2024.2418909)
Supplement: Table S3 Bayes factor for host transmission of H4 viruses in the Eurasian lineage.docx [file TEMI_A_2418909_SM8386.docx]

**Table S3**. Bayes factor for host transmission of H4 viruses in the Eurasian lineage.

| **From** | **To** | **Bayes factor** | **Posterior probability** |
| --- | --- | --- | --- |
| Domestic Anseriformes | Wild Anseriformes | 174141.2088 | 1 |
| Wild Anseriformes | Ciconiiformes | 174141.2088 | 0.9999 |
| Domestic Anseriformes | Domestic Galliformes | 14506.0114 | 0.9995 |
| Wild Anseriformes | Charadriiformes | 13389.6814 | 0.9995 |
| Wild Anseriformes | Domestic Anseriformes | 28.2054 | 0.8179 |
| Domestic Anseriformes | Swine | 26.3265 | 0.8074 |
| Domestic Anseriformes | Seal | 7.1944 | 0.5339 |
