# Supplementary material for: Epidemiology and biological characteristics of influenza A (H4N6) viruses from wild birds
Source: Emerg Microbes Infect. 2024 Oct 17;13(1):2418909. doi: 10.1080/22221751.2024.2418909 (PMC11523250; doi:10.1080/22221751.2024.2418909)
Supplement: Figure S4 The genotypes of nine H4N6 isolates from wild birds.pdf [file TEMI_A_2418909_SM8385.pdf]

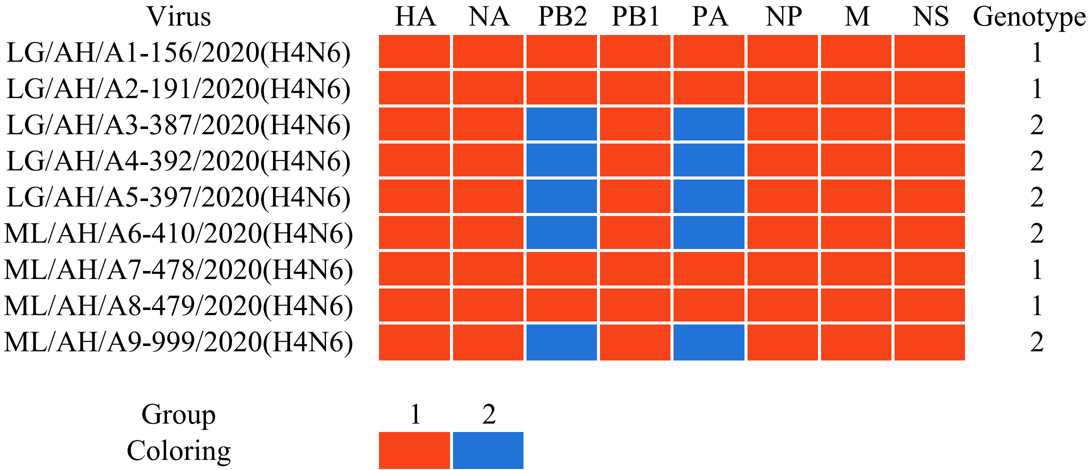
Virus
HA
NA
PB2
PB1
PA
NP
M
NS
Genotype
LG/AH/A1-156/2020(H4N6)
1
LG/AH/A2-191/2020(H4N6)
1
LG/AH/A3-387/2020(H4N6)
2
LG/AH/A4-392/2020(H4N6)
2
LG/AH/A5-397/2020(H4N6)
2
ML/AH/A6-410/2020(H4N6)
2
ML/AH/A7-478/2020(H4N6)
1
ML/AH/A8-479/2020(H4N6)
1
ML/AH/A9-999/2020(H4N6)
2
Group
Coloring
1
2
