# Supplementary material for: Epidemiology and biological characteristics of influenza A (H4N6) viruses from wild birds
Source: Emerg Microbes Infect. 2024 Oct 17;13(1):2418909. doi: 10.1080/22221751.2024.2418909 (PMC11523250; doi:10.1080/22221751.2024.2418909)
Supplement: Table S5 The highest nucleotide homology of the whole genomes of two representative H4N6 viruses.docx [file TEMI_A_2418909_SM8384.docx]

**Table S5**. The highest nucleotide homology of the whole genomes of two representative H4N6 viruses.

| **Virus name** | **Segment** | **Highest homologous strain** |  |  |  |
| --- | --- | --- | --- | --- | --- |
|  |  | **Name** | **Abbreviation** | **Homology** | **Accession** |
| LG/AH/A1-156/  2020(H4N6) | PB2 | A/duck/Mongolia/826/2019(H4N6) | DK/MN/826/2019(H4N6) | 99.21% | MT020259.1 |
|  | PB1 | A/duck/Mongolia/826/2019(H4N6) | DK/MN/826/2019(H4N6) | 99.34% | MT020260.1 |
|  | PA | A/spot-billed duck/South Korea/JB32-105/2019(H4N2) | SBD/SKO/JB32-105/2019(H4N2) | 99.63% | MW494118.1 |
|  | HA | A/duck/Bangladesh/41653/2019(H4N2) | DK/BD/41653/2019(H4N2) | 99.59% | MW466370.1 |
|  | NP | A/wild duck/South Korea/#57/2020(H7N7) | WD/SKO/#57/2020(H7N7) | 99.13% | OK175684.1 |
|  | NA | A/Mallard (Anas platyrhynchos)/South Korea/KNU2019-61/2019(H4N6) | ML/SKO/KNU2019-61/2019(H4N6) | 99.22% | MW391692.1 |
|  | M | A/common teal/Shanghai/JDS110203/2019(H12N8) | CT/SH/JDS110203/2019(H12N8) | 99.90% | MN795770.1 |
|  | NS | A/wild goose/dongting lake/121/2018(H6N2) | WG/DTL/121/2018(H6N2) | 99.64% | MH727484.1 |
| ML/AH/A9-999/  2020(H4N6) | PB2 | A/duck/Vietnam/HN5894/2019(H4N6) | DK/VN/HN5894/2019(H4N6) | 97.59% | MW935149.1 |
|  | PB1 | A/duck/Mongolia/826/2019(H4N6) | DK/MN/826/2019(H4N6) | 99.25% | MT020260.1 |
|  | PA | A/Mallard(Anas platyrhynchos)/South Korea/KNU2019-54/2019(H5N3) | ML/SKO/KNU2019-54/2019(H5N3) | 99.21% | MW386776.1 |
|  | HA | A/mallard/South Korea/JB17-85/2019(H4N6) | ML/SKO/JB17-85/2019(H4N6) | 99.59% | MW494140.1 |
|  | NP | A/wild duck/South Korea/#57/2020(H7N7) | WD/SKO/#57/2020(H7N7) | 99.06% | OK175684.1 |
|  | NA | A/Mallard (Anas platyrhynchos)/South Korea/KNU2019-61/2019(H4N6) | ML/SKO/KNU2019-61/2019(H4N6) | 99.29% | MW391692.1 |
|  | M | A/duck/Mongolia/961/2019(H3N8) | DK/MN/961/2019(H3N8) | 99.39% | MT020281.1 |
|  | NS | A/wild goose/dongting lake/121/2018(H6N2) | WG/DTL/121/2018(H6N2) | 99.52% | MH727484.1 |
